# Supplementary material for: Distinct filament morphology and membrane tethering features of the dual FtsZ paralogs in Odinarchaeota
Source: EMBO J. Author manuscript; Available in PMC 2025 Nov 15. (PMC12583498; doi:10.1038/s44318-025-00529-7)
Supplement: Appendix [file EMS209695-supplement-Appendix.pdf]

Appendix for

Distinct filament morphology and membrane tethering features of the dual FtsZs in Odinarchaeota

Jayanti Kumari[1*], Akhilesh Uthaman[1*], Sucharita Bose[2#], Ananya Kundu[3,4#], Vaibhav Sharma[5#], Soumyajit Dutta[6], Anubhav Dhar[1], Srijita Roy[7,8], Ramanujam Srinivasan[7,8], Samay Pande[5], Kutti R. Vinothkumar[3], Pananghat Gayathri[6]✉, Saravanan Palani[1]✉

✉gayathri@iiserpune.ac.in (PG); spalani@iisc.ac.in (SP)

Table of contents:

Appendix Figure S1.. .3
Appendix Figure S2..4-5
Appendix Figure S3...6
Appendix Table S1..7

Appendix Figure S1

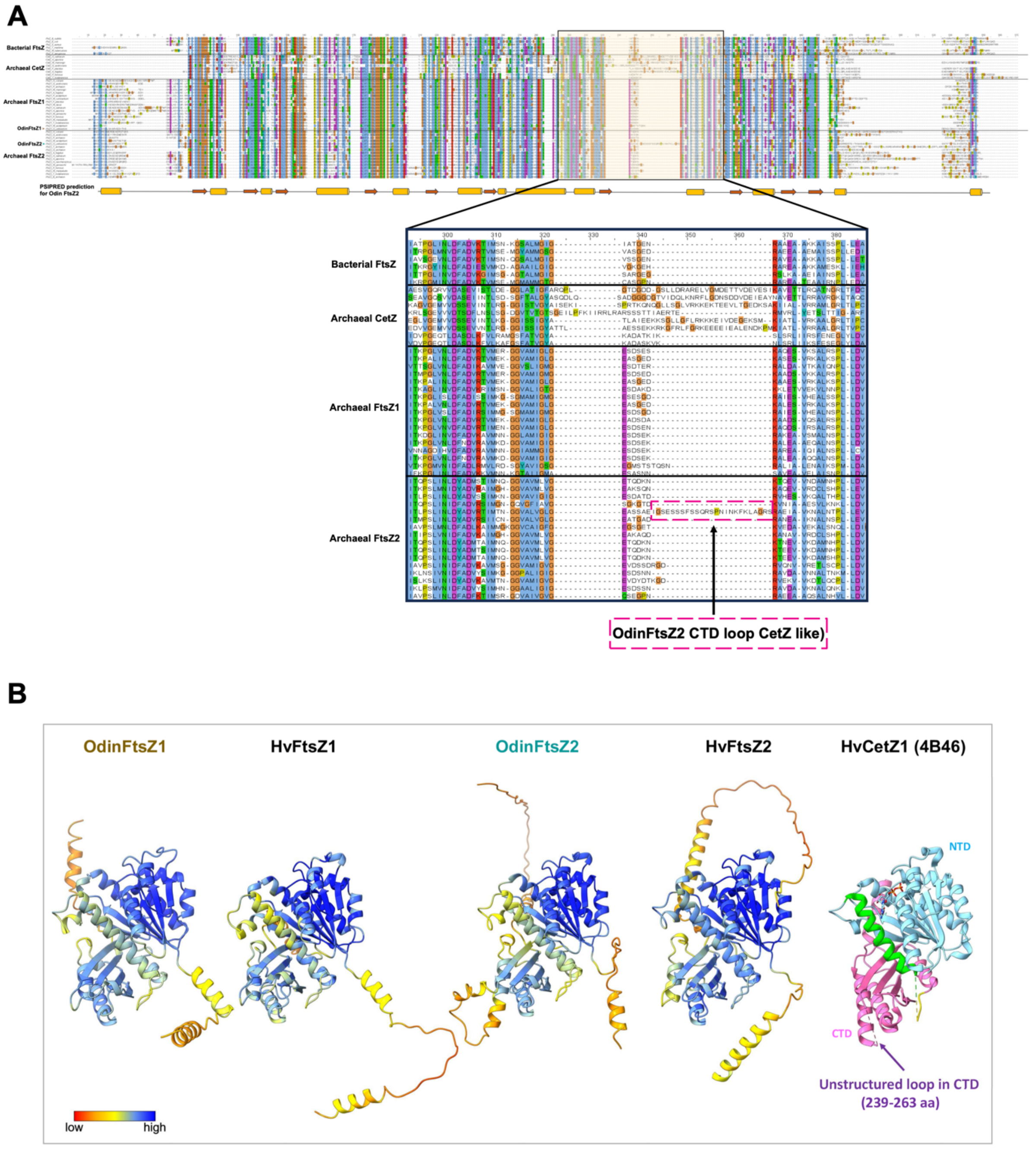

Appendix Figure S1: A CetZ like loop insertion is present in OdinFtsZ2

(A) Multiple sequence alignment of representative FtsZ sequences from bacterial, archaeal CetZ and archaeal FtsZs showing CetZ-like loop insertion unique to OdinFtsZ2 (adapted from (Liao *et al*, 2021)). **(B)** Protomer structure comparison of OdinFtsZ1 and OdinFtsZ2 monomers with *Haloferax volcanii* (HvFtsZ1 (UniProt Accession no: Q48327) HvFtsZ2 (UniProt Accession no: D4GSH7), and HvCetZ (PDB ID: 4B46)). The cartoon representation of the AlphaFold structures is colored according to the pLDDT score from AlphaFold2, with red and blue shades representing low and high respectively, with a gradation as shown with the color bar.

Appendix Figure S2

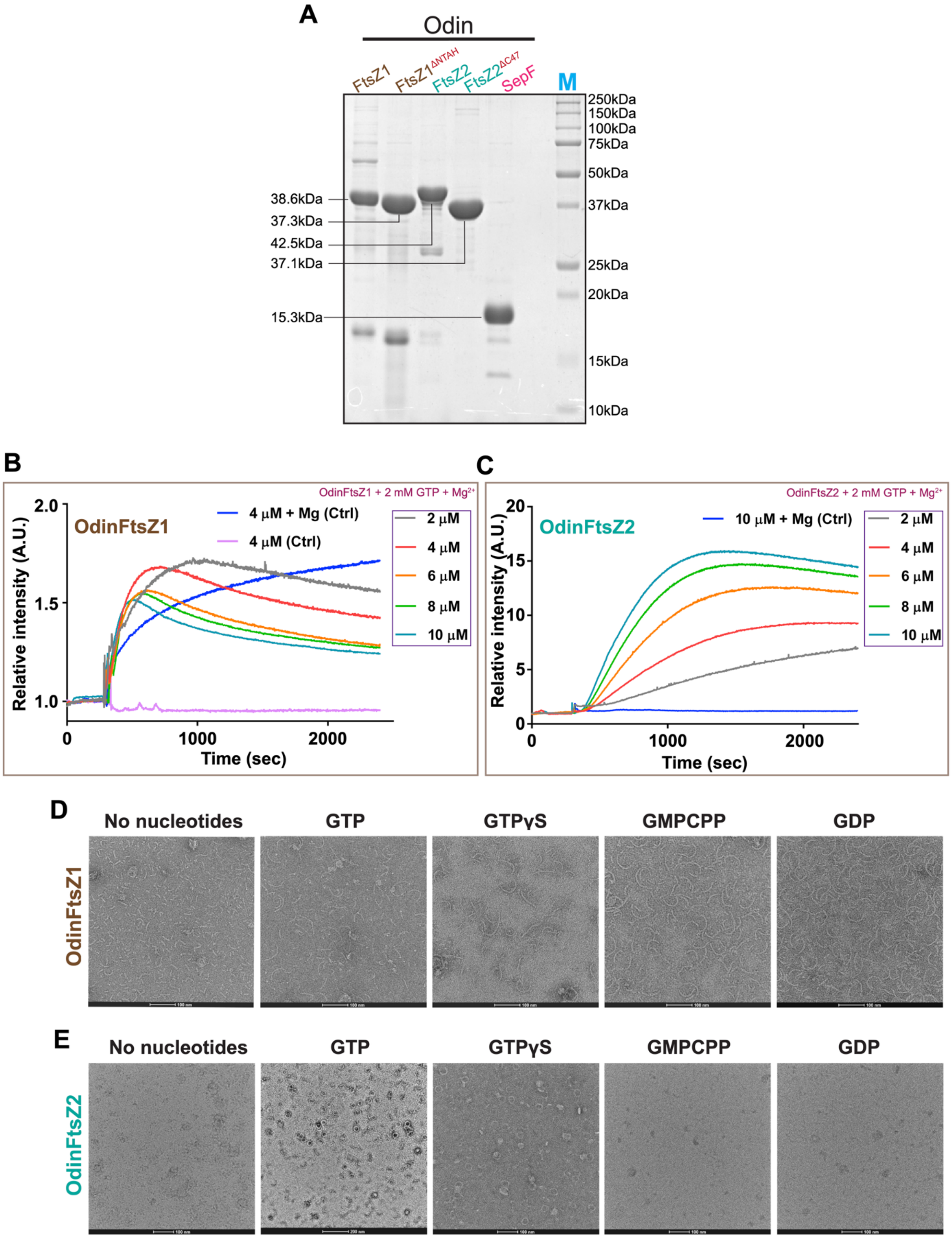

Appendix Figure S2: Differential scattering profile and filament assembly by OdinFtsZ1 and OdinFtsZ2 proteins

(A) Representative 12% SDS-PAGE gel for all the untagged proteins used in the study. The protein bands are labeled with their expected sizes. **(B)** The plot of relative light scattering intensity (y-axis) with time (x-axis) for different concentrations of OdinFtsZ1 protein polymerized in the presence of 2 mM GTP and 5 mM Mg^{2+}. The relative scattering intensity of the protein at 4 μM with Mg^{2+} (in blue) and without Mg^{2+} (in magenta) but devoid of any nucleotide acts as a control. **(C)** The plot of relative light scattering intensity (y-axis) with time (x-axis) for OdinFtsZ2 protein polymerized in the presence of 2 mM GTP and 5 mM Mg^{2+}. The blue curve represents 10 μM protein polymerized solely in the presence of 5 mM Mg^{2+}, which serves as the control. **(D)** Negative stain micrographs of 0.04 mg/ml OdinFtsZ1 supplemented with (a) no nucleotide, (b) 2 mM GTP, (c) 2 mM GTPγS, (d) 2 mM GMPCPP and (e) 2 mM GDP. The scale bar represents 100 nm. **(E)** Negative stain micrographs of 0.1 mg/ml OdinFtsZ2 supplemented with (a) no nucleotide, (b) 2 mM GTP (scale bar -200 nm), (c) 2 mM GTPγS, (d) 2 mM GMPCPP and (e) 2 mM GDP. scale bar -100 nm. These results are replicated over at least two independent experiments. The scale bar represents 100 nm unless mentioned otherwise.

Appendix Figure S3

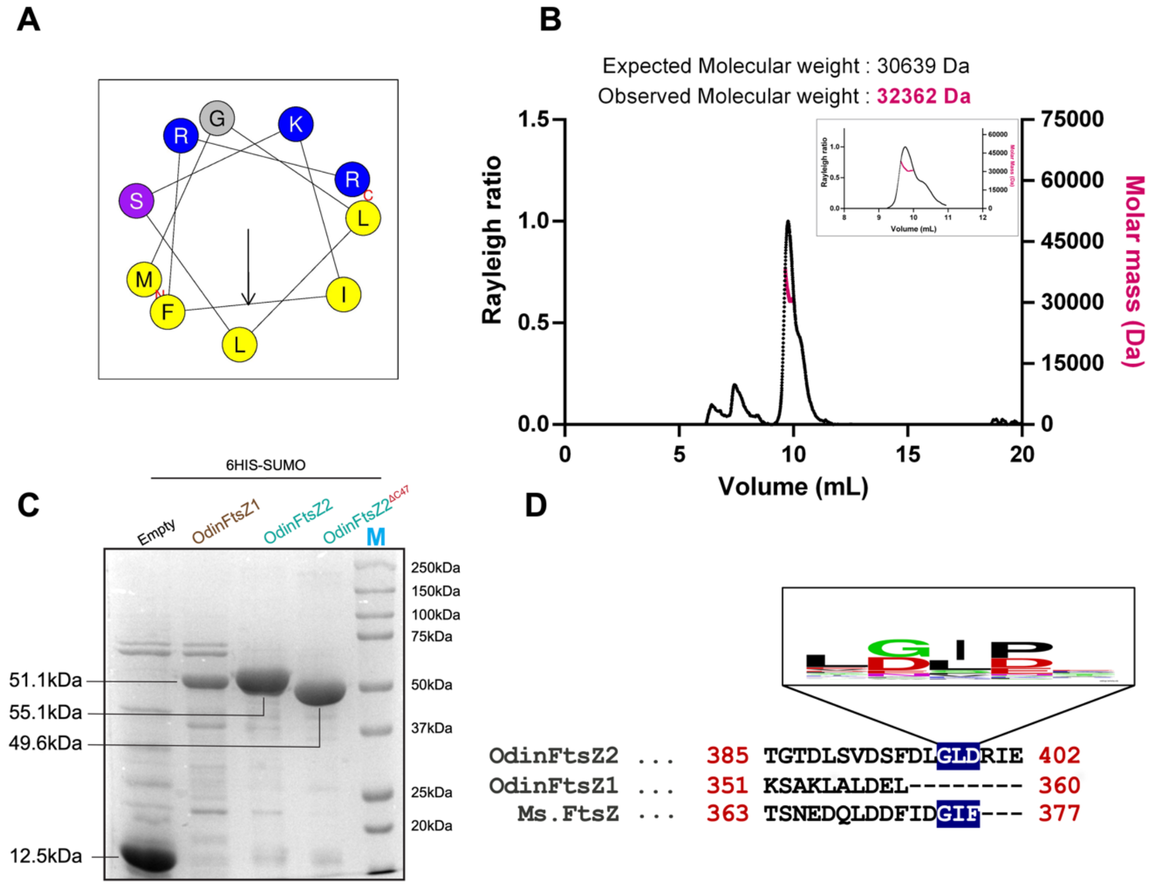

Appendix Figure S3: SepF, an archaeal membrane anchor protein

(A) Representative helical wheel diagram for the predicted amphipathic helix (AH) at the N-terminus of OdinSepF screened using Heliquest (version 1.2 Analysis module). **(B)** SEC-MALS profile of purified OdinSepF. The chromatogram displays the calculated molar mass of the peaks (Da) and Rayleigh ratio as red and black lines, respectively. **(C)** Representative 15% SDS-PAGE gel for proteins used in pull-down experiments shown in panels (Fig. 3C, Fig. 3D). **(D)** Sequence logo for the conservation of the GID motif in the C-terminal tail of FtsZ proteins. The logo was created using WebLogo (link) based on a multiple sequence alignment of 69 FtsZ sequences used in the preparation of the phylogenetic tree described in Fig. EV1. The alignment shown in the figure has three representative sequence namely OdinFtsZ1 (UniProt Accession: A0A1Q9N645), OdinFtsZ2 (UniProt Accession: A0A1Q9N6K6) and *Methanobrevibacter smithii*; Ms.FtsZ (UniProt Accession: A0A2H4U4M7).

Appendix Table S1: List of plasmids used in this study

Plasmid Number	Description	Source
piSP65	pET28a-6HIS-bdSUMO (Empty)	(Hatano *et al*, 2022)
piSP1749	pET28a-6HIS-bdSUMO-OdinFtsZ1	This Study
piSP1751	pET28a-6HIS-bdSUMO-OdinFtsZ2	This Study
piSP1753	pET28a-6HIS-bdSUMO-OdinSepF	This Study
piSP1780	pET28a-6HIS-bdSUMO-OdinFtsZ1$^{\Delta NTAH}$(14-363a.a)	This Study
piSP1786	pET28a-6HIS-bdSUMO-OdinFtsZ2$^{\Delta C47}$ (1-355a.a)	This Study

References:

Hatano T, Palani S, Papatziamou D, Salzer R, Souza DP, Tamarit D, Makwana M, Potter A, Haig A, Xu W, *et al* (2022) Asgard archaea shed light on the evolutionary origins of the eukaryotic ubiquitin-ESCRT machinery. *Nat Commun* 13: 3398

Liao Y, Ithurbide S, Evenhuis C, Löwe J & Duggin IG (2021) Cell division in the archaeon Haloferax volcanii relies on two FtsZ proteins with distinct functions in division ring assembly and constriction. *Nat Microbiol* 6: 594–605
