# Supplementary material for: Distinct filament morphology and membrane tethering features of the dual FtsZ paralogs in Odinarchaeota
Source: EMBO J. Author manuscript; Available in PMC 2025 Nov 15. (PMC12583498; doi:10.1038/s44318-025-00529-7)
Supplement: Expanded View Figures [file EMS209695-supplement-Expanded_View_Figures.pdf]

Expanded View Figures

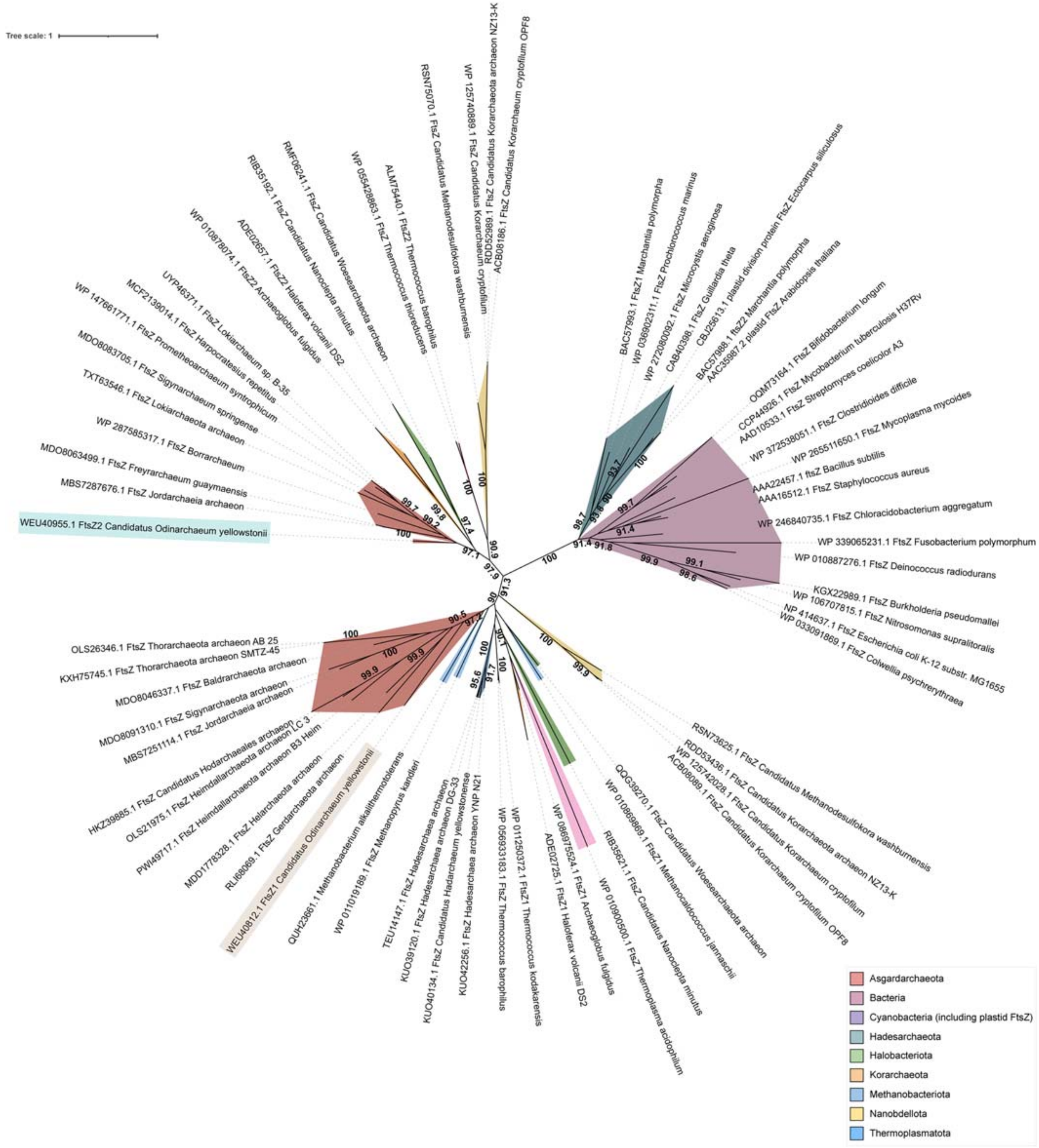

Figure EV1. Maximum likelihood (ML) tree of representative FtsZ sequences.

The unrooted ML tree was generated for FtsZ using the protein sequences from bacteria and archaea. The phylogenetic tree was generated using IQTREE with model LG + G4 + I (69 sequences, 396 amino acid sites). Source data are available online for this figure.

© The Author(s)

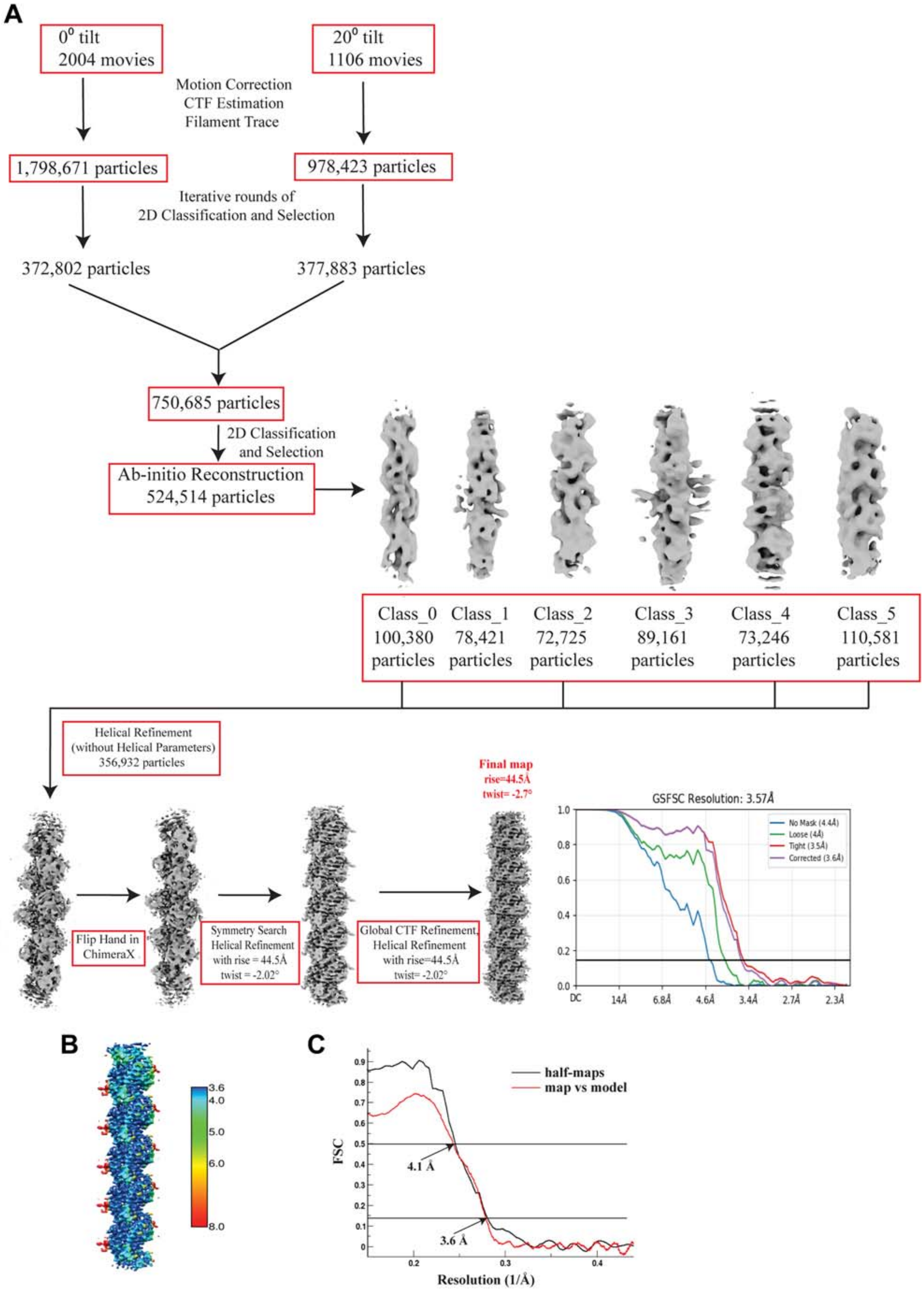
A
0° tilt
2004 movies
20° tilt
1106 movies
Motion Correction
CTF Estimation
Filament Trace
1,798,671 particles
978,423 particles
Iterative rounds of
2D Classification and Selection
372,802 particles
377,883 particles
750,685 particles
2D Classification
and Selection
Ab-initio Reconstruction
524,514 particles
Class_0 100,380 particles
Class_1 78,421 particles
Class_2 72,725 particles
Class_3 89,161 particles
Class_4 73,246 particles
Class_5 110,581 particles
Helical Refinement
(without Helical Parameters)
356,932 particles
Flip Hand in ChimeraX
Symmetry Search
Helical Refinement
with rise = 44.5Å
twist = -2.02°
Global CTF Refinement,
Helical Refinement
with rise=44.5Å
twist= -2.02°
Final map
rise=44.5Å
twist= -2.7°
GSFSC Resolution: 3.57Å
No Mask (4.4Å)
Loose (4Å)
Tight (3.5Å)
Corrected (3.6Å)
DC
14Å
6.8Å
4.6Å
3.4Å
2.7Å
2.3Å
B
3.6
4.0
5.0
6.0
8.0
C
half-maps
map vs model
FSC
4.1 Å
3.6 Å
Resolution (1/Å)

Figure EV2. Cryo-EM data processing workflow of OdinFtsZ1 single filament.

(**A**) Two datasets were collected, untilted and another with a 20° tilt and processed individually till 2D classification and then the best classes were combined. These classes were processed in cryoSPARC, and the workflow is described. The GSFSC curves from cryoSPARC are shown with different masks and an estimated overall resolution of 3.6 Å. (**B**) The local resolution plot of the map shows the core of the filament resolved better and the periphery poorly resolved as expected. (**C**) Comparison of the FSC's derived from the half maps (@ 0.143, black curve) and map vs model (@ 0.5, red curve) indicates a resolution of 3.6 and 4.1 Å, respectively. Source data are available online for this figure.

 © The Author(s)

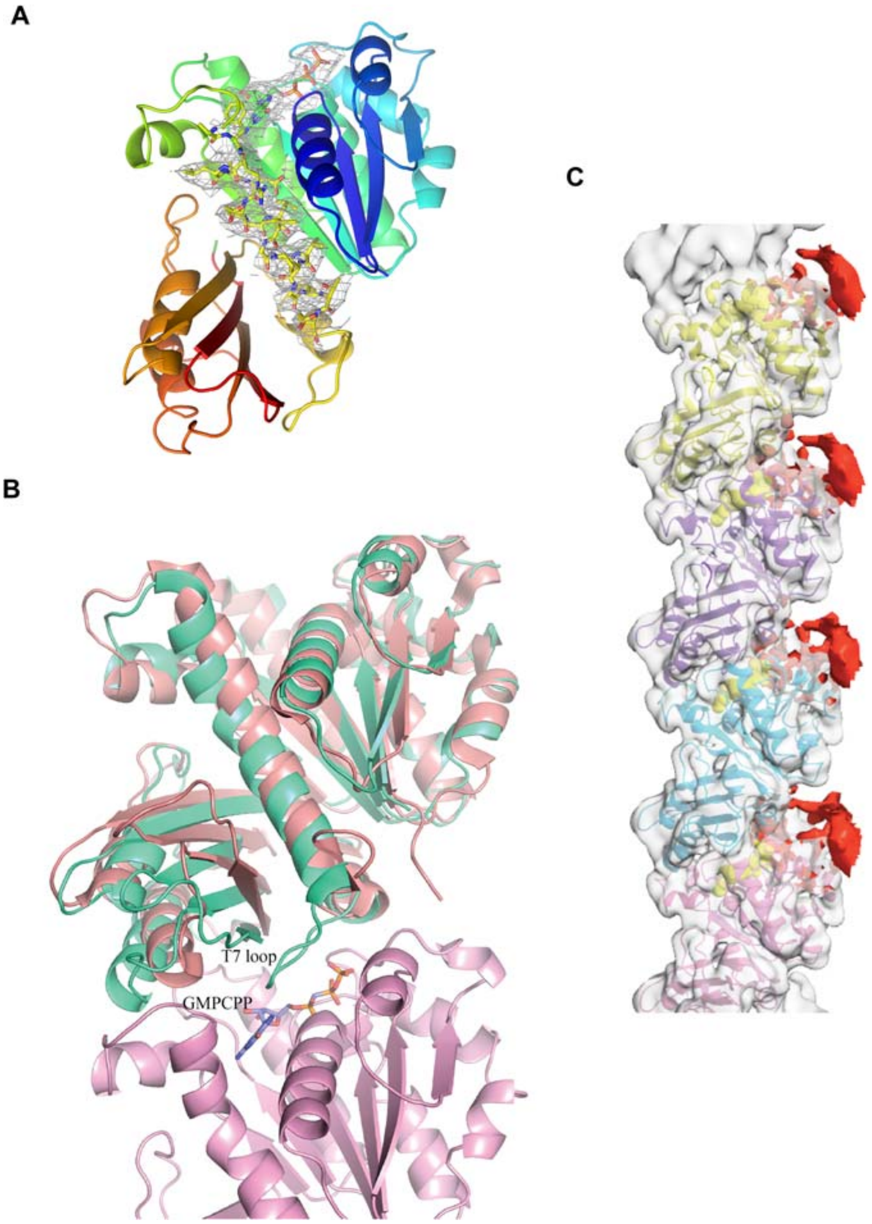

Figure EV3. OdinFtsZ1 model fit into the final sharpened map.

(**A**) The OdinFtsZ1 monomer is shown in cartoon representation and colored in rainbow from the N-terminus (blue) to the C-terminus (red). The central helix (H7) and GMPCPP are represented as sticks and encased in the final_sym_sharpened map contoured at ~7σ in PyMol. (**B**) OdinFtsZ1 is present in T-conformation in the filaments. Superimposition of OdinFtsZ1 (monomer A, salmon) on *Staphylococcus aureus* (SaFtsZ) protein in R conformation (PDB ID: 5H5G, Chain B), (green) shows that the central helix is pushed one turn down with the catalytic T7 loop inserted into the subunit interface which is in close proximity to the GMPCPP molecule bound to the second monomer (monomer B, pink). (**C**) Unmodelled density at the N-terminus of the OdinFtsZ1 monomer. Although residues 34–332 have been modeled, additional density (red) is visible at the N-terminus, suggesting the presence of unresolved residues. The map quality in this region is low to allow for unambiguous modeling. Source data are available online for this figure.

© The Author(s)

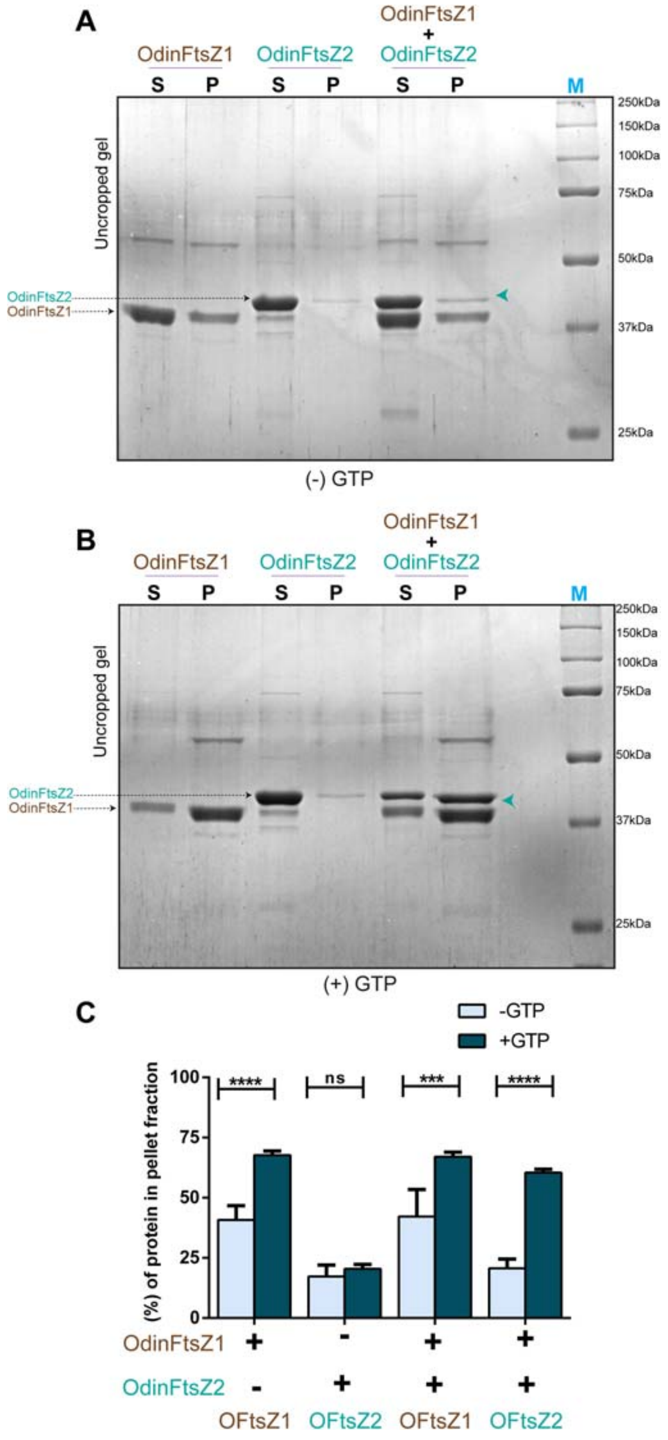

Figure EV4. OdinFtsZ1 and OdinFtsZ2 co-pellet on ultracentrifugation.

(**A**) Representative 12% SDS-PAGE gel for sedimentation of individual proteins OdinFtsZ1 (10 μM), OdinFtsZ2 (10 μM) and the co-sedimentation when incubated in equimolar concentration (10 μM) in the absence of GTP. (**B**) Representative 12% SDS-PAGE gel for sedimentation of individual proteins OdinFtsZ1 (10 μM), OdinFtsZ2 (10 μM) and the co-sedimentation when incubated in equimolar concentration (10 μM) in the presence of GTP nucleotide. Both supernatant (S) and pellet (P) fractions upon ultracentrifugation at 100,000×*g* were loaded. (**C**) Representative plot for the mean percentage of protein in the pellet fraction (*y* axis) with and without GTP. The pellet (or supernatant) fraction intensities corresponding to the band were calculated as the intensity of the pellet (or supernatant) divided by the sum of the two intensities and represented in the graph as a percentage. These percentages are represented as mean ± standard error of the mean (SEM) in the graph. '+' or '−' below the bars denote the presence/absence of Odin FtsZs, while the OFtsZ1/OFtsZ2 labeled below the bottom lines denote the protein band which has been quantified. ($N = 3$ (results replicated over three independent experiment), Two-way ANOVA with Sidak's multiple comparisons test was used, ns -non-significant, ***$P = 0.0001572$, ****$P = 0.0000638$ (for OdinFtsZ1), ****$P = 0.0000005$ (for OdinFtsZ2(+Z1))). Source data are available online for this figure.

 © The Author(s)

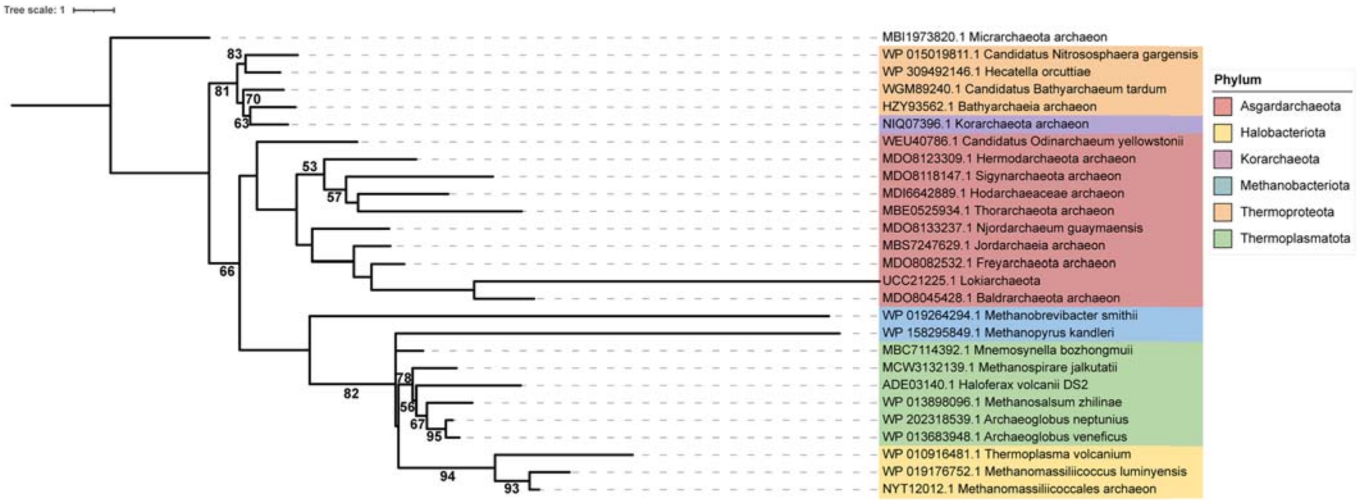

Figure EV5. SepF as a universal archaeal membrane anchor protein.

The ML tree for representatives archaeal SepF sequences, generated using IQTREE with LG + G4 + C60 model (27 sequences, 118 amino acid sites). The tree was rooted using Micrarchaeota (DPANN group) as an outgroup. Source data are available online for this figure.
