# Supplementary material for: Distinct filament morphology and membrane tethering features of the dual FtsZ paralogs in Odinarchaeota
Source: EMBO J. 2025 Aug 8;44(21):4. doi: 10.1038/s44318-025-00529-7 (PMC12583498; doi:10.1038/s44318-025-00529-7)

SepF - Liposome Copelleting

Replicate 1

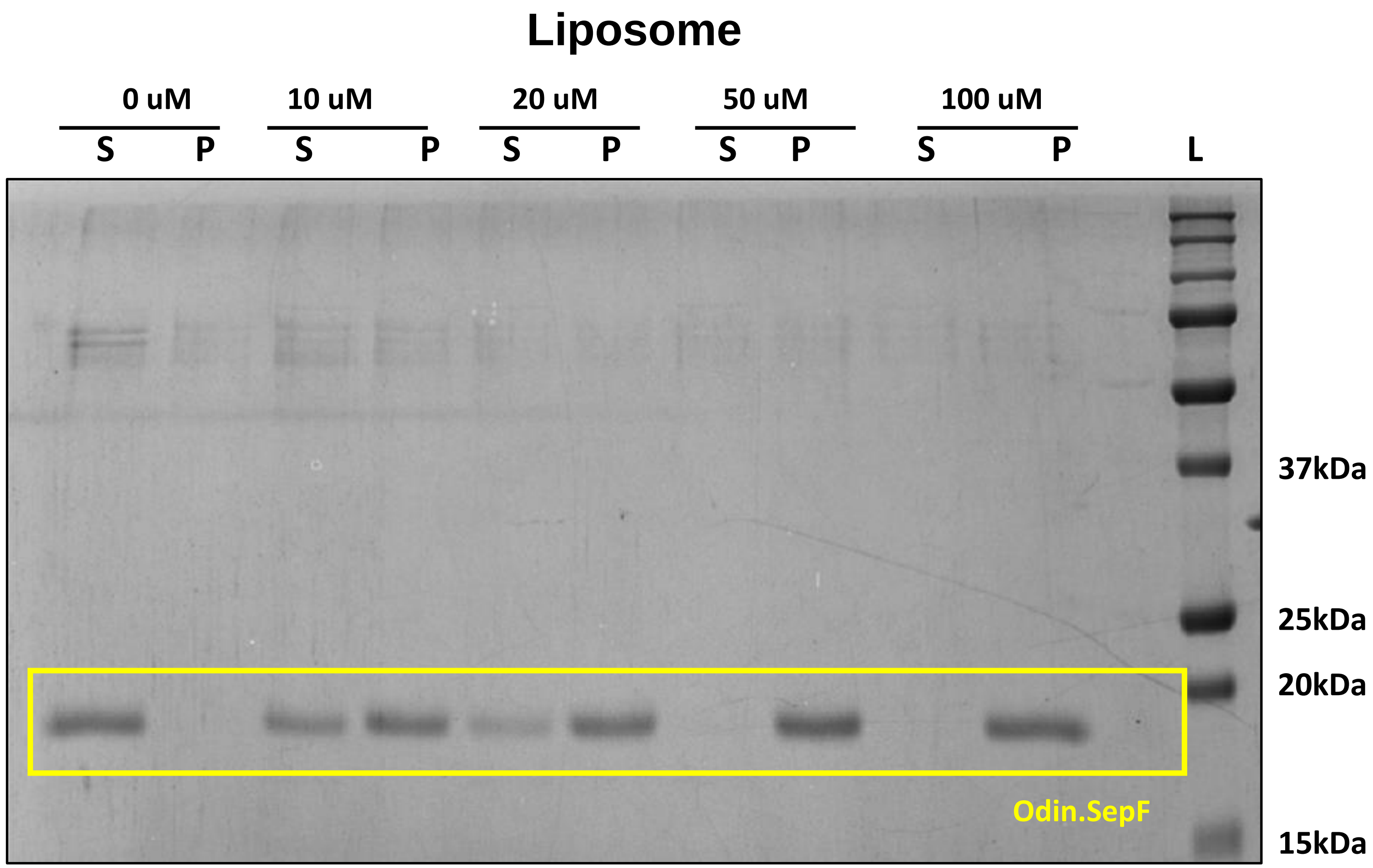

Replicate 2

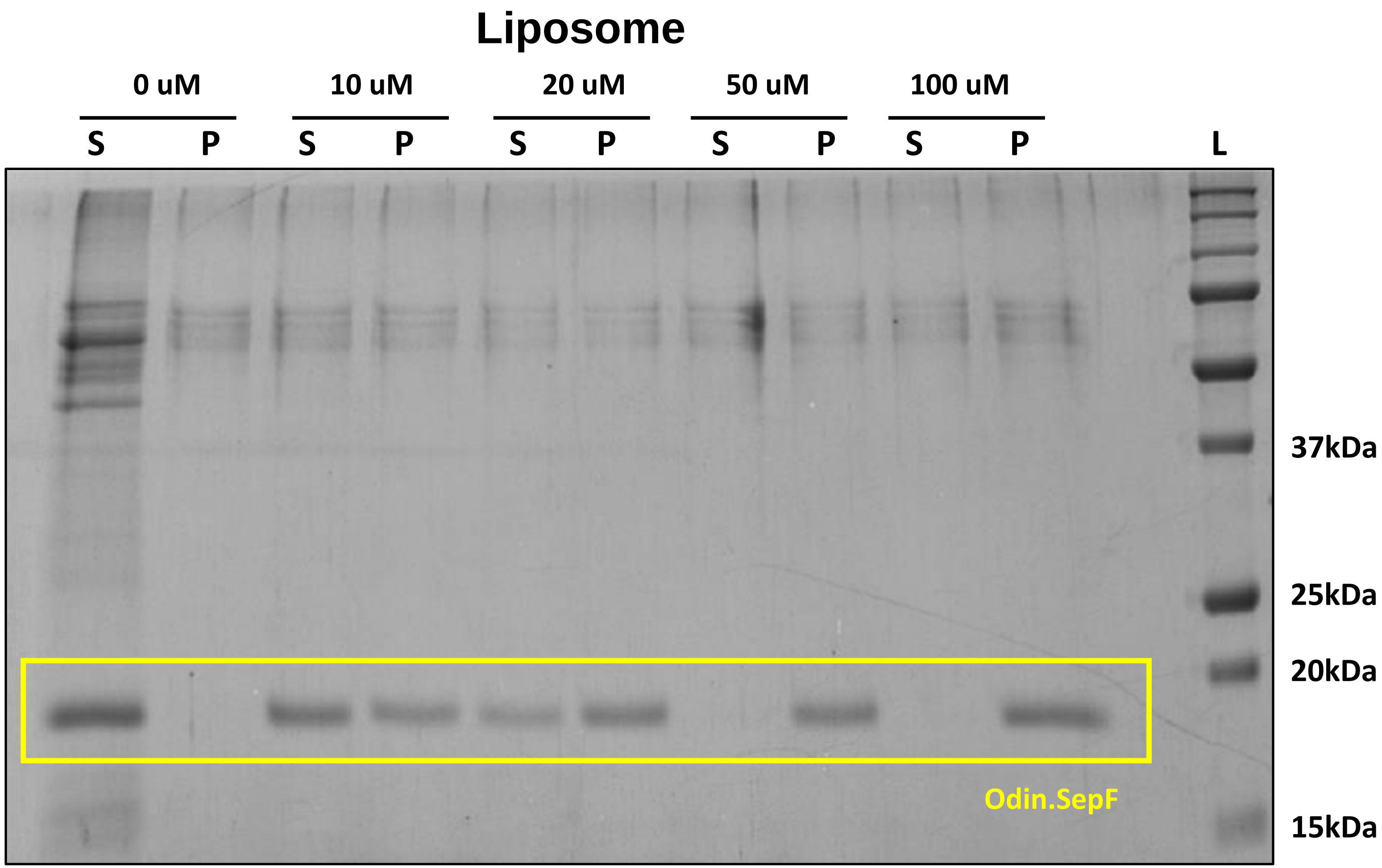
Liposome
0 uM
10 uM
20 uM
50 uM
100 uM
S P S P S P S P S P
L
37kDa
25kDa
20kDa
15kDa
Odin.SepF

Replicate 3

Liposome

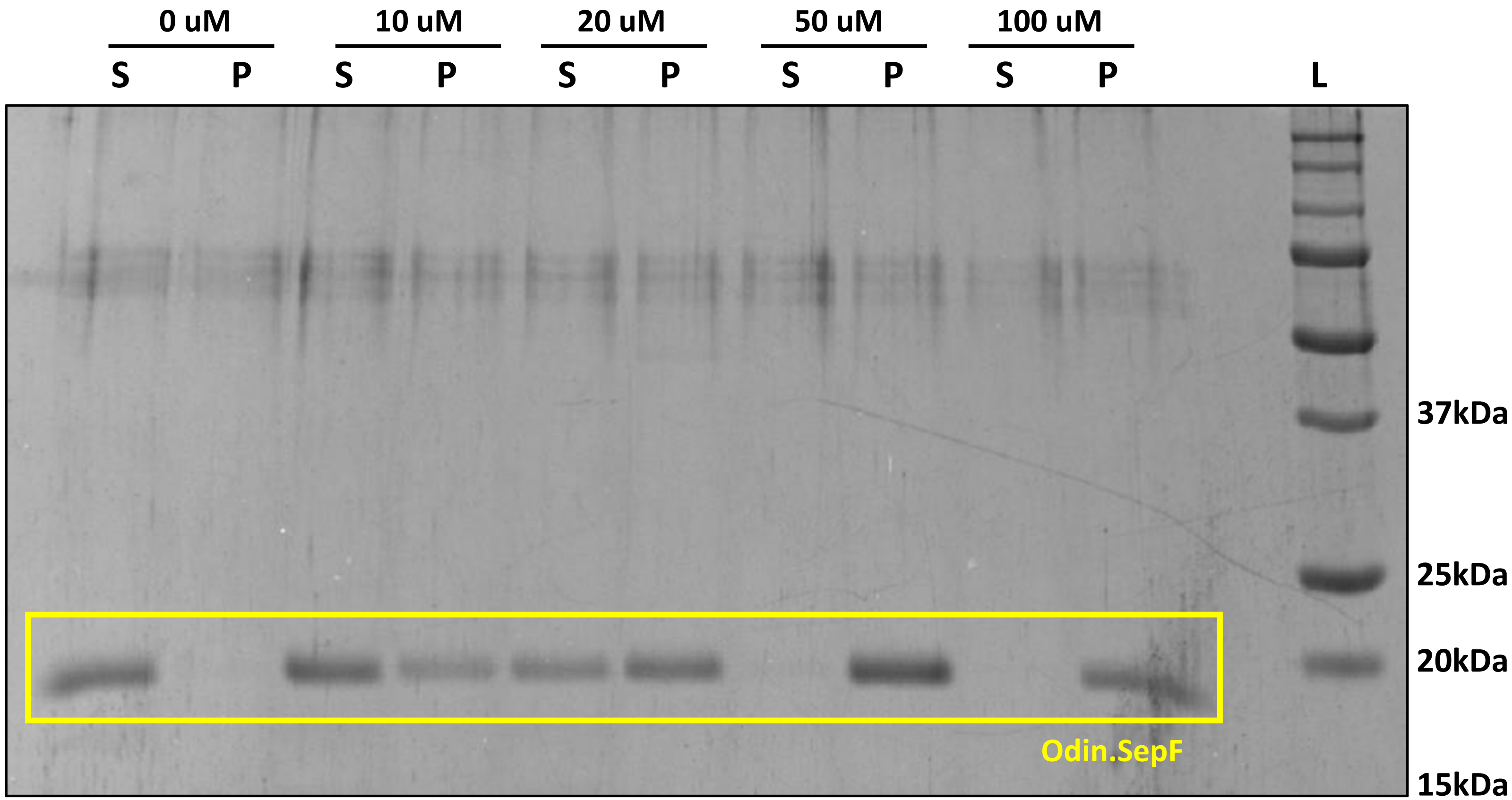

Supplement: Supplementary file 3 [file 44318_2025_529_MOESM5_ESM.zip › Figure 3 /Fig. 3A/Fig. 3A_README.pdf]
