# Supplementary material for: Distinct filament morphology and membrane tethering features of the dual FtsZ paralogs in Odinarchaeota
Source: EMBO J. 2025 Aug 8;44(21):4. doi: 10.1038/s44318-025-00529-7 (PMC12583498; doi:10.1038/s44318-025-00529-7)
Supplement: Supplementary file 3 [file 44318_2025_529_MOESM5_ESM.zip › Figure 3 /Fig. 3E/Fig. 3E_README.pdf]

Replicate 1

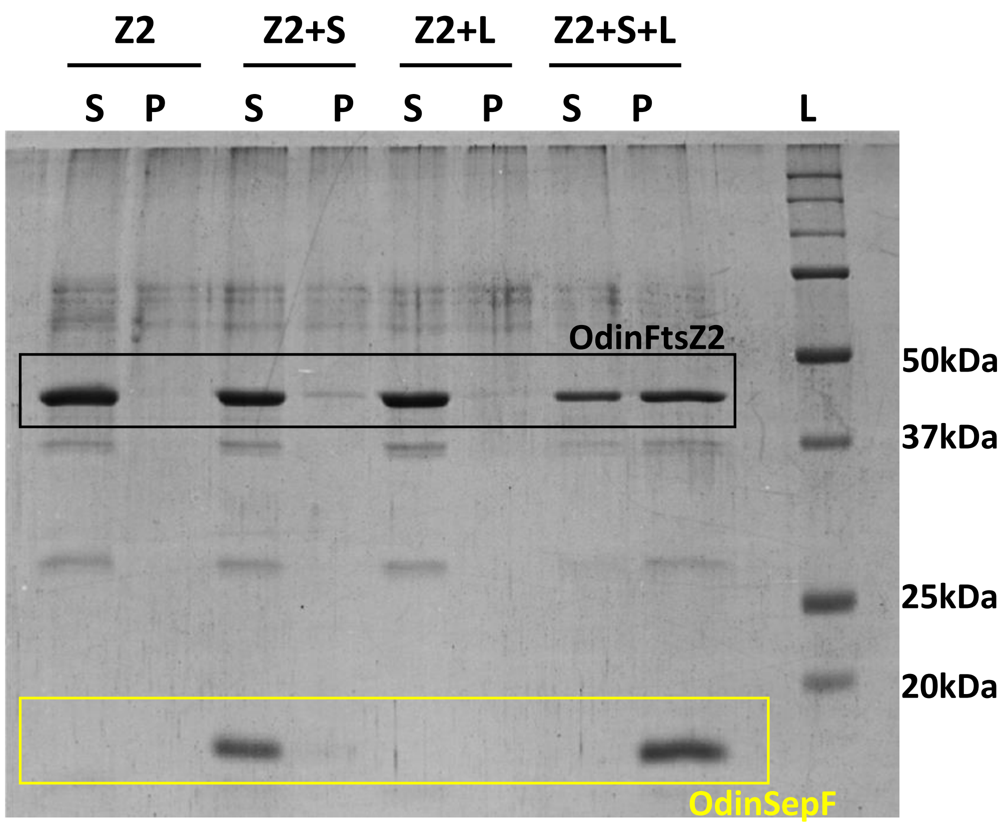

L - 600 uM DOPG
Z2 - 2 uM OdinFtsZ2
S - 2 uM OdinSepF

Replicate 2

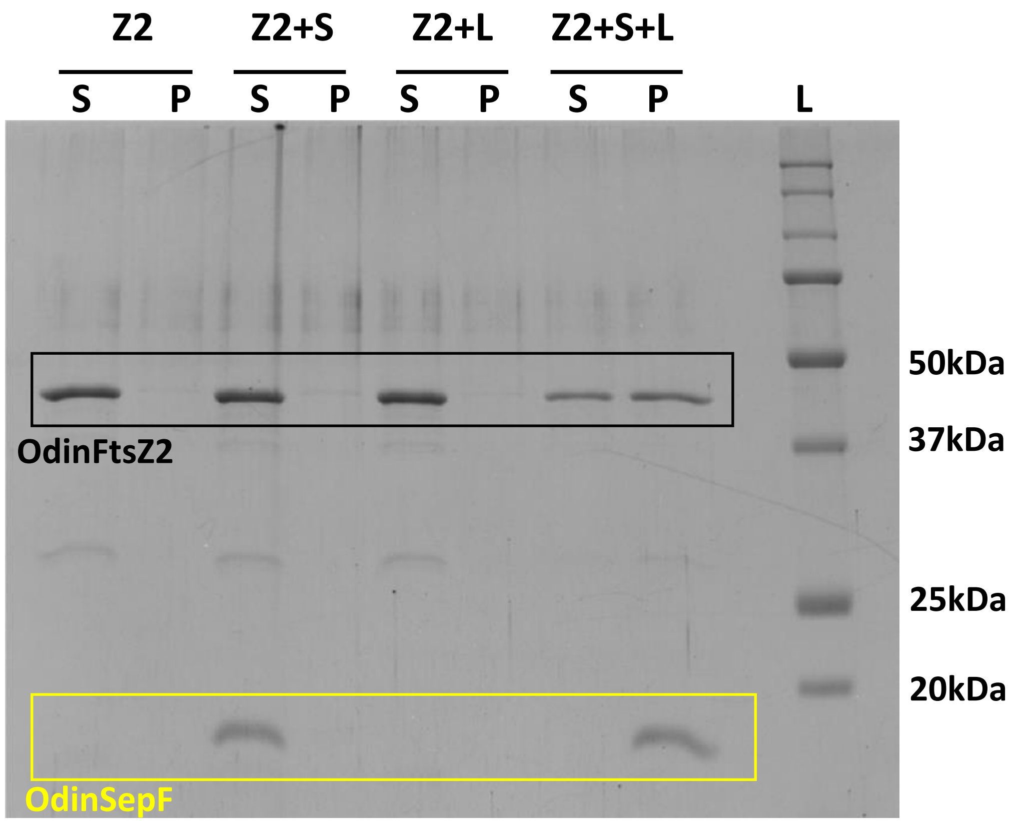

L - 600 uM DOPG
Z2 - 2 uM OdinFtsZ2
S - 2 uM OdinSepF

Replicate 3

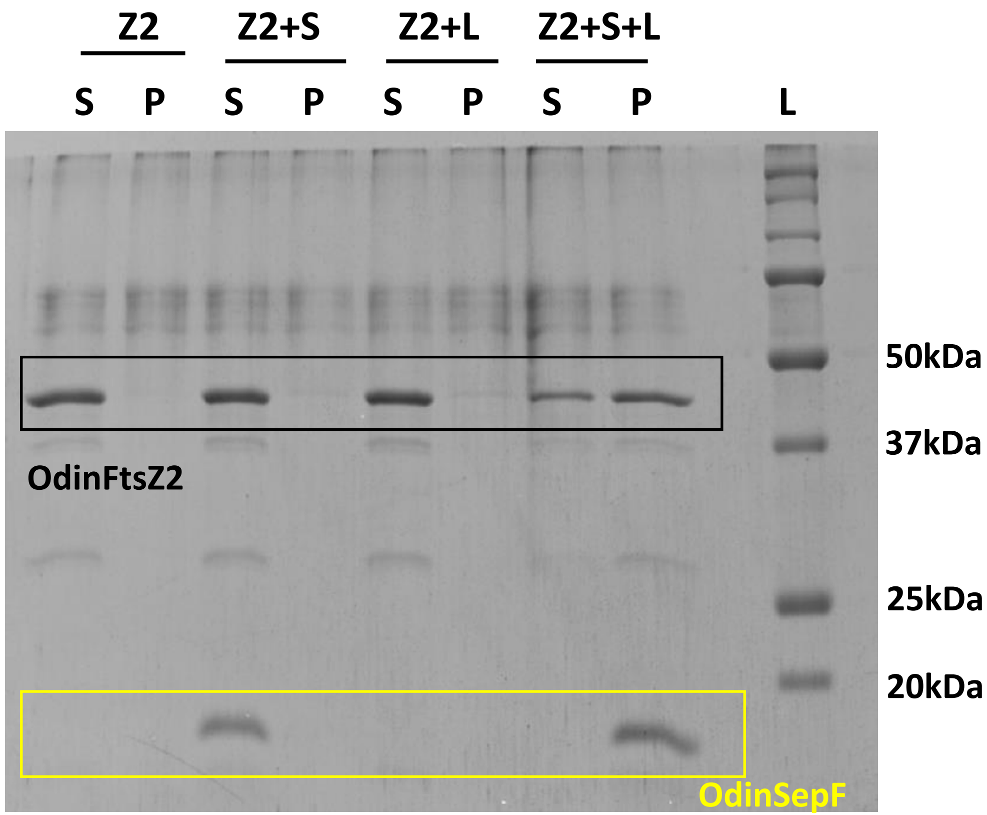

L - 600 uM DOPG
Z2 - 2 uM OdinFtsZ2
S - 2 uM OdinSepF
