# Supplementary material for: Distinct filament morphology and membrane tethering features of the dual FtsZ paralogs in Odinarchaeota
Source: EMBO J. 2025 Aug 8;44(21):4. doi: 10.1038/s44318-025-00529-7 (PMC12583498; doi:10.1038/s44318-025-00529-7)
Supplement: Supplementary file 3 [file 44318_2025_529_MOESM5_ESM.zip › Figure 3 /Fig. 3D/Fig. 3D_README.pdf]

Replicate 1

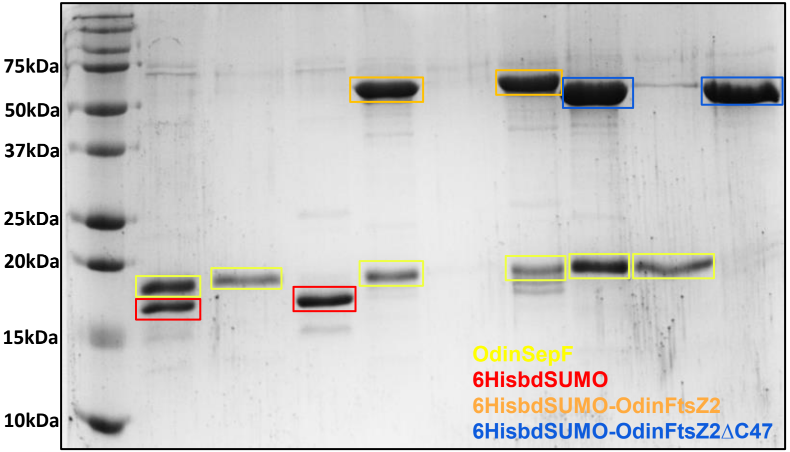

Replicate 2

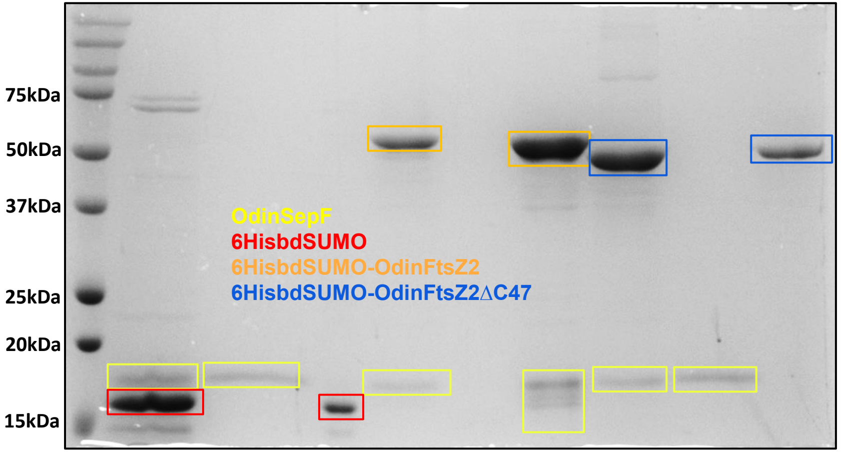

Replicate 3

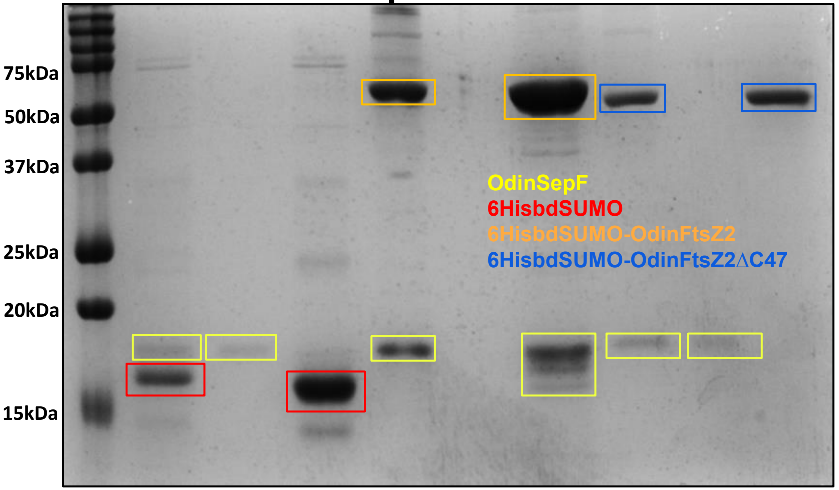

Yellow Box- OdinSepF
Red Box- 6HisbdSUMO
Orange Box- 6HisbdSUMO-OdinFtsZ2
Blue Box- 6HisbdSUMO-OdinFtsZ2ΔC47
