# Supplementary material for: Distinct filament morphology and membrane tethering features of the dual FtsZ paralogs in Odinarchaeota
Source: EMBO J. 2025 Aug 8;44(21):4. doi: 10.1038/s44318-025-00529-7 (PMC12583498; doi:10.1038/s44318-025-00529-7)
Supplement: Supplementary file 3 [file 44318_2025_529_MOESM5_ESM.zip › Figure 3 /Fig. 3C/Fig. 3C_README.pdf]

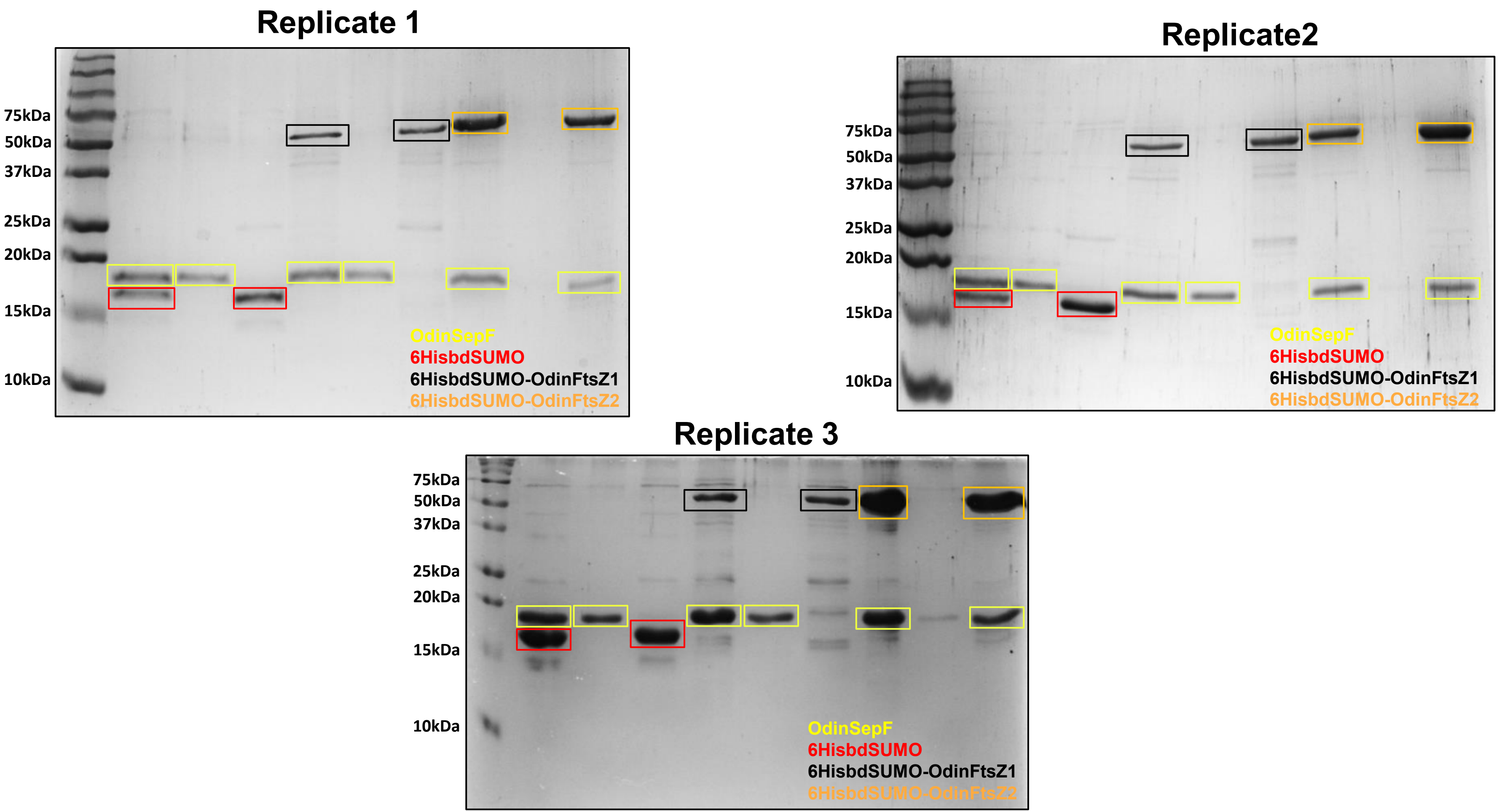
Replicate 1
75kDa
50kDa
37kDa
25kDa
20kDa
15kDa
10kDa
OdinSepF
6HisbdSUMO
6HisbdSUMO-OdinFtsZ1
6HisbdSUMO-OdinFtsZ2
Replicate2
75kDa
50kDa
37kDa
25kDa
20kDa
15kDa
10kDa
OdinSepF
6HisbdSUMO
6HisbdSUMO-OdinFtsZ1
6HisbdSUMO-OdinFtsZ2
Replicate 3
75kDa
50kDa
37kDa
25kDa
20kDa
15kDa
10kDa
OdinSepF
6HisbdSUMO
6HisbdSUMO-OdinFtsZ1
6HisbdSUMO-OdinFtsZ2
