# Supplementary material for: Distinct filament morphology and membrane tethering features of the dual FtsZ paralogs in Odinarchaeota
Source: EMBO J. 2025 Aug 8;44(21):4. doi: 10.1038/s44318-025-00529-7 (PMC12583498; doi:10.1038/s44318-025-00529-7)
Supplement: Supplementary file 4 [file 44318_2025_529_MOESM6_ESM.zip › Figure 4/Fig. 4D/Fig. 4D_README.pdf]

# Liposome Co-pelleting assay for OdinFtsZ1 ΔNTD1

## Replicate 1

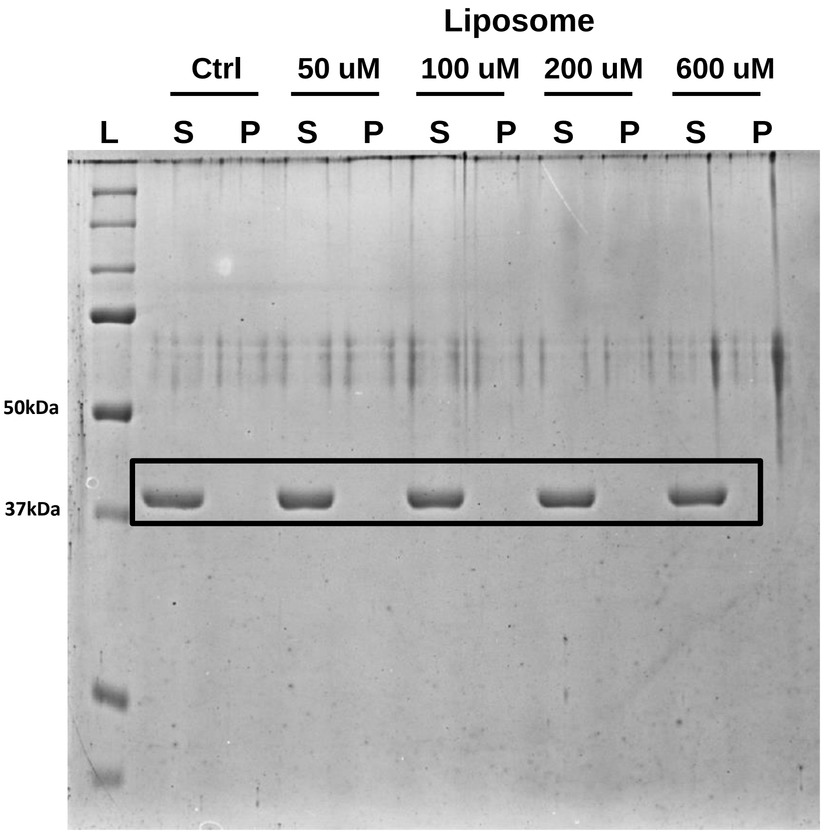

# Replicate 2

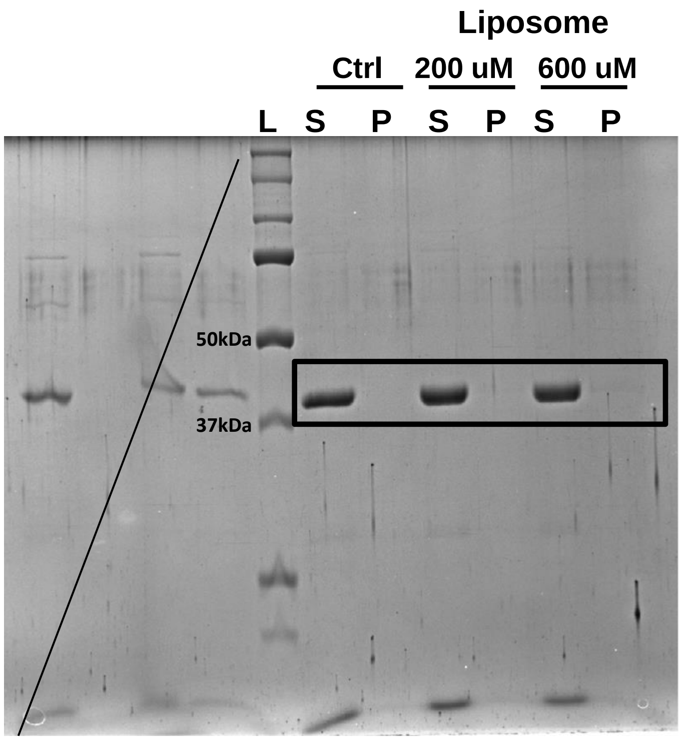


Del NTD

# Replicate 3

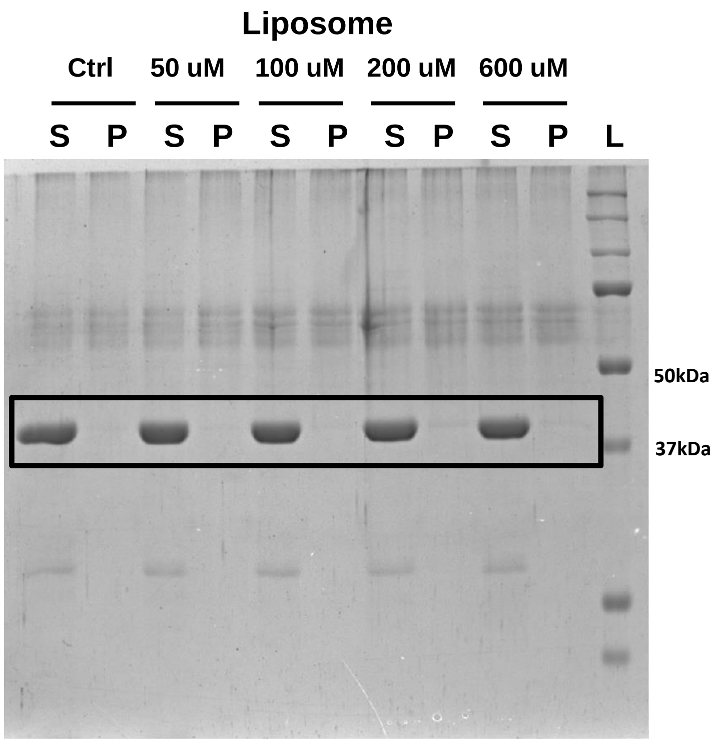

Liposome
Ctrl
50 uM
100 uM
200 uM
600 uM
S
P
S
P
S
P
S
P
S
P
L
50kDa
37kDa
