# Supplementary material for: Distinct filament morphology and membrane tethering features of the dual FtsZ paralogs in Odinarchaeota
Source: EMBO J. 2025 Aug 8;44(21):4. doi: 10.1038/s44318-025-00529-7 (PMC12583498; doi:10.1038/s44318-025-00529-7)

Liposome Co-pelleting assay for OdinFtsZ1

Replicate 3

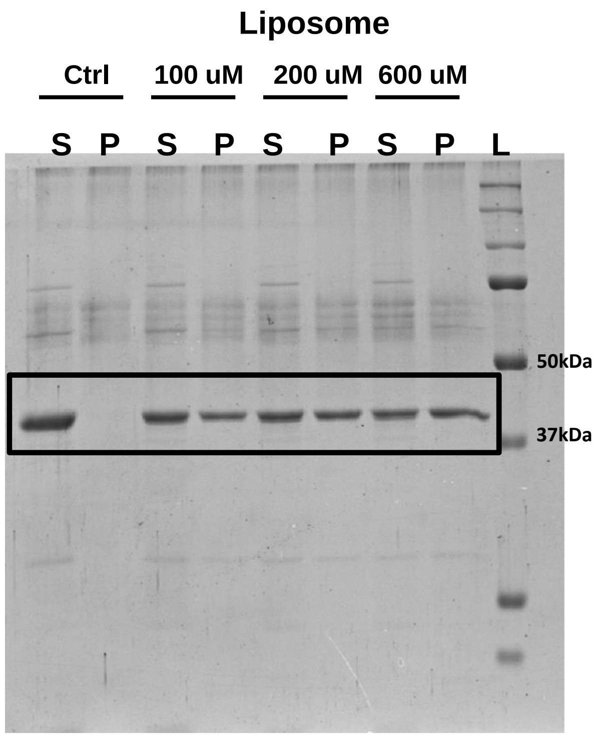

Replicate 1

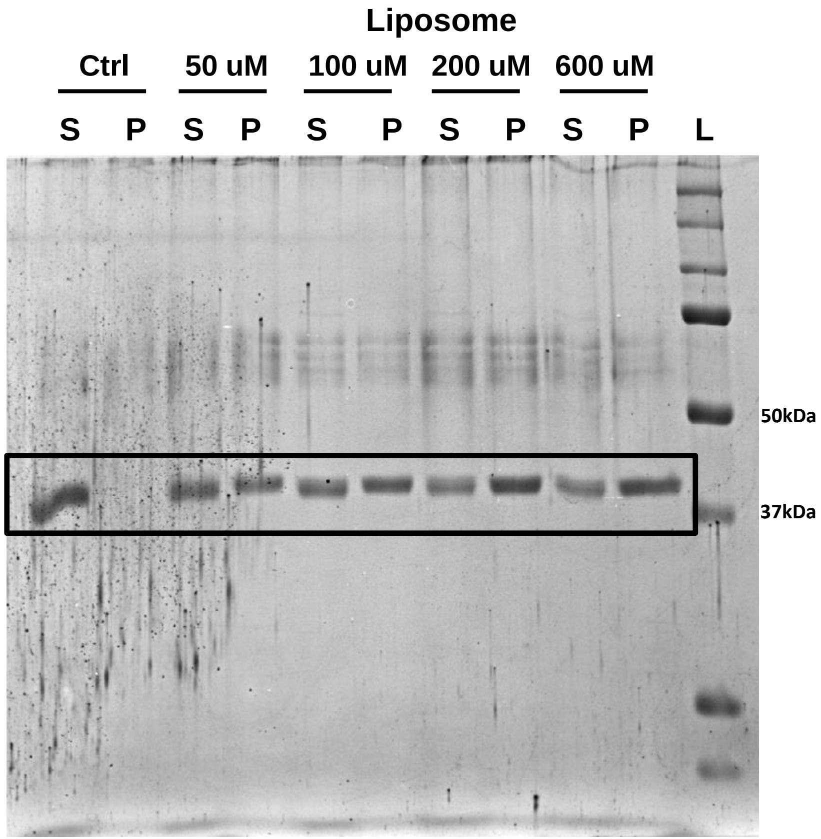
Liposome
Ctrl
50 uM
100 uM
200 uM
600 uM
S P S P S P S P S P L
50kDa
37kDa

Replicate 2

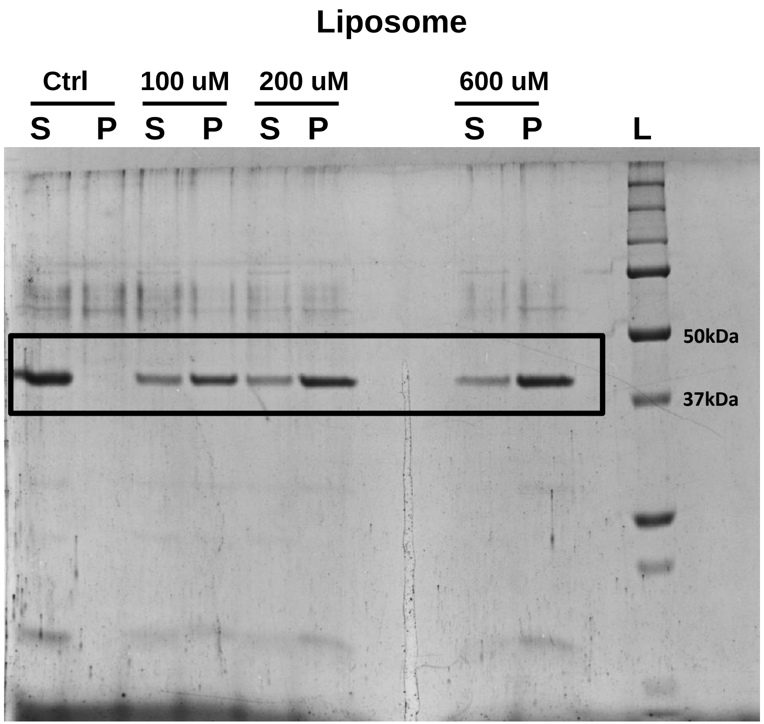

Supplement: Supplementary file 4 [file 44318_2025_529_MOESM6_ESM.zip › Figure 4/Fig. 4B/Fig. 4B_README.pdf]
