# Supplementary material for: Distinct filament morphology and membrane tethering features of the dual FtsZ paralogs in Odinarchaeota
Source: EMBO J. 2025 Aug 8;44(21):4. doi: 10.1038/s44318-025-00529-7 (PMC12583498; doi:10.1038/s44318-025-00529-7)

Replicate 2_MAIN

Co-pelleting assay between OdinFtsZ1 and OdinFtsZ2 (-GTP)

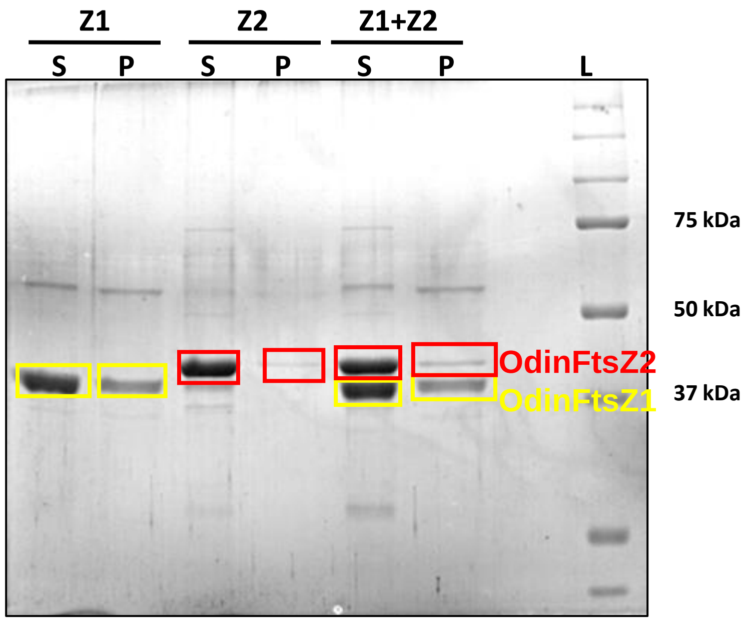

Replicate 1

Co-pelleting assay between OdinFtsZ1 and OdinFtsZ2 (-GTP)

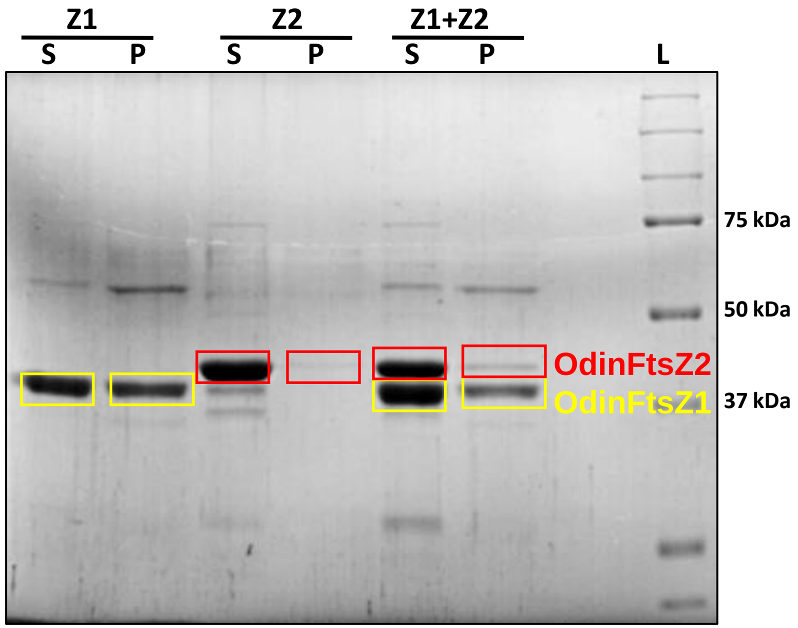

Replicate 3

Co-pelleting assay between OdinFtsZ1 and OdinFtsZ2 (-GTP)

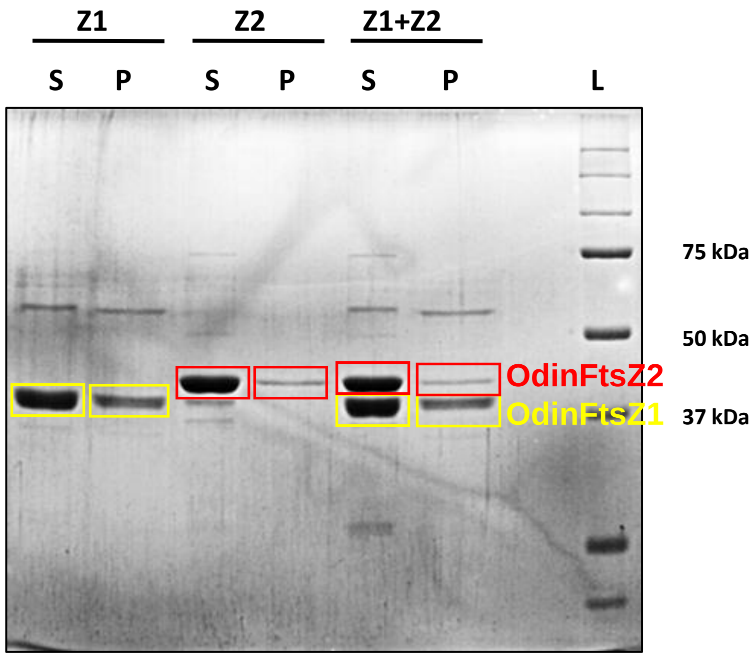

Supplement: Supplementary file 5 [file 44318_2025_529_MOESM7_ESM.zip › Expanded view Figures/Figure EV4/Fig. EV4A/Fig. EV4A_README.pdf]
