# Supplementary material for: Distinct filament morphology and membrane tethering features of the dual FtsZ paralogs in Odinarchaeota
Source: EMBO J. 2025 Aug 8;44(21):4. doi: 10.1038/s44318-025-00529-7 (PMC12583498; doi:10.1038/s44318-025-00529-7)
Supplement: Supplementary file 5 [file 44318_2025_529_MOESM7_ESM.zip › Expanded view Figures/Figure EV1/Fig. EV1/Fig. EV1.pdf]

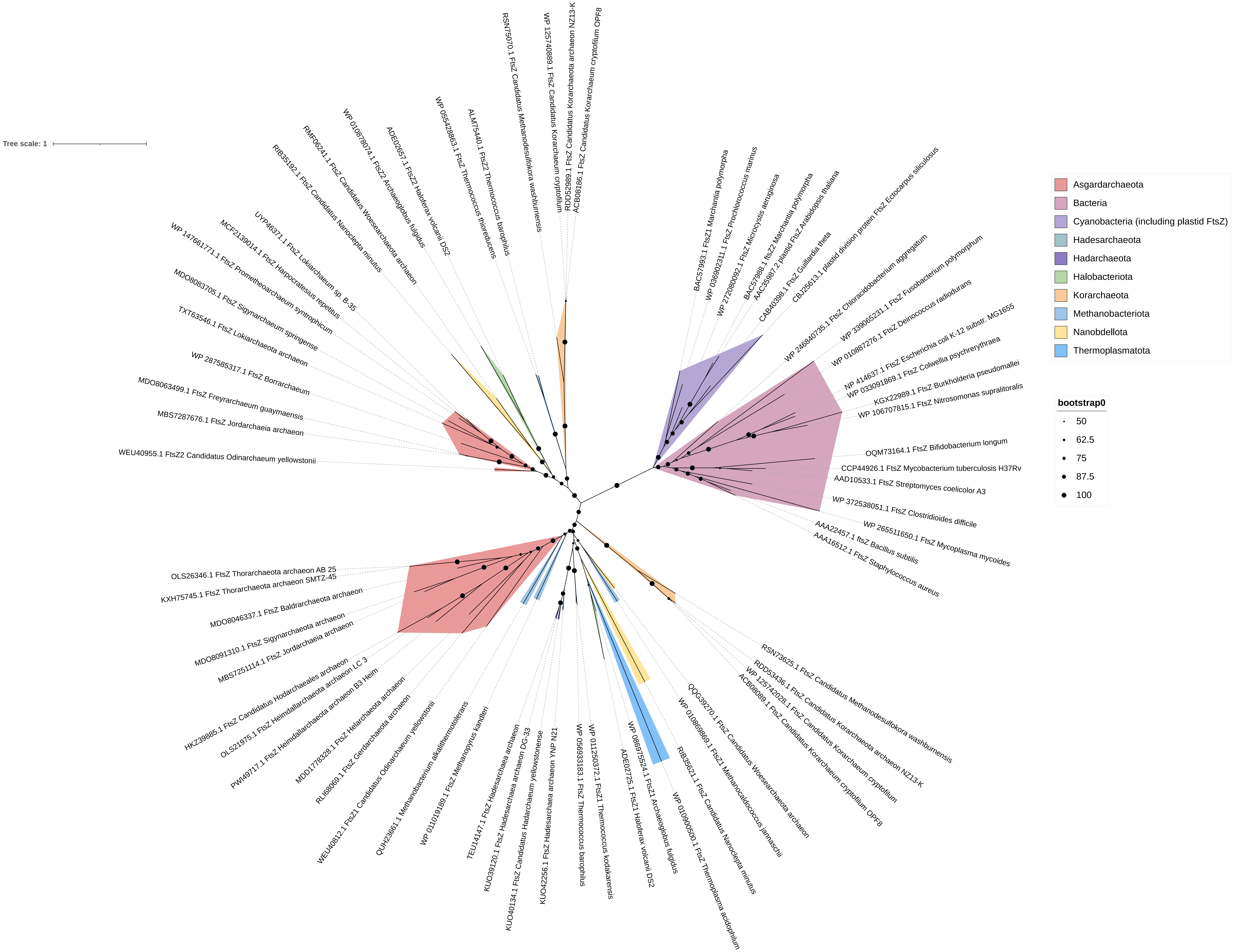
Tree scale: 1
Asgardarchaeota
Bacteria
Cyanobacteria (including plastid FtsZ)
Hadesarchaeota
Hadarchaeota
Halobacteriota
Korarchaeota
Methanobacteriota
Nanobdellota
Thermoplasmatota
bootstrap0
50
62.5
75
87.5
100
RSN75070.1 FtsZ Candidatus Methanodesulfokora washburnensis
WP 125740889.1 FtsZ Candidatus Korarchaeum cryptofilum
RDD52989.1 FtsZ Candidatus Korarchaeota archaeon NZ13-K
ACB08186.1 FtsZ Candidatus Korarchaeum cryptofilum OPF8
ALM75440.1 FtsZ2 Thermococcus barophilus
WP 055428863.1 FtsZ Thermococcus thioreducens
ADE02657.1 FtsZ2 Haloferax volcanii DS2
WP 010878074.1 FtsZ2 Archaeoglobus fulgidus
RMF06241.1 FtsZ Candidatus Woesearchaeota archaeon
RIB35192.1 FtsZ Candidatus Nanoclepta minutus
UYP46371.1 FtsZ Lokiarchaeum sp. B-35
MCF2139014.1 FtsZ Harpocratesius repetitus
WP 147661771.1 FtsZ Prometheoarchaeum syntrophicum
MDO8083705.1 FtsZ Sigynarchaeum springense
TXT63546.1 FtsZ Lokiarchaeota archaeon
WP 287585317.1 FtsZ Borrarchaeum
MDO8063499.1 FtsZ Freyrarchaeum guaymaensis
MBS7287676.1 FtsZ Jordarchaeia archaeon
WEU40955.1 FtsZ2 Candidatus Odinarchaeum yellowstonii
BAC57993.1 FtsZ1 Marchantia polymorpha
WP 036902311.1 FtsZ Prochlorococcus marinus
WP 272080092.1 FtsZ Microcystis aeruginosa
BAC57988.1 ftsZ2 Marchantia polymorpha
AAC35987.2 plastid FtsZ Arabidopsis thaliana
CAB40398.1 FtsZ Guillardia theta
CBJ25613.1 plastid division protein FtsZ Ectocarpus siliculosus
WP 246840735.1 FtsZ Chloracidobacterium aggregatum
WP 339065231.1 FtsZ Fusobacterium polymorphum
WP 010887276.1 FtsZ Deinococcus radiodurans
NP 414637.1 FtsZ Escherichia coli K-12 substr. MG1655
WP 033091869.1 FtsZ Colwellia psychrerythraea
KGX22989.1 FtsZ Burkholderia pseudomallei
WP 106707815.1 FtsZ Nitrosomonas supralitoralis
OQM73164.1 FtsZ Bifidobacterium longum
CCP44926.1 FtsZ Mycobacterium tuberculosis H37Rv
AAD10533.1 FtsZ Streptomyces coelicolor A3
WP 372538051.1 FtsZ Clostridioides difficile
WP 265511650.1 FtsZ Mycoplasma mycoides
AAA22457.1 ftsZ Bacillus subtilis
AAA16512.1 FtsZ Staphylococcus aureus
OLS26346.1 FtsZ Thorarchaeota archaeon AB 25
KXH75745.1 FtsZ Thorarchaeota archaeon SMTZ-45
MDO8046337.1 FtsZ Baldrarchaeota archaeon
MDO8091310.1 FtsZ Sigynarchaeota archaeon
MBS7251114.1 FtsZ Jordarchaeia archaeon
HKZ39885.1 FtsZ Candidatus Hodarchaeales archaeon
OLS21975.1 FtsZ Heimdallarchaeota archaeon LC 3
PWI49717.1 FtsZ Heimdallarchaeota archaeon B3 Heim
MDD1778328.1 FtsZ Helarchaeota archaeon
RLI68069.1 FtsZ Gerdarchaeota archaeon
WEU40812.1 FtsZ1 Candidatus Odinarchaeum yellowstonii
QUH23661.1 Methanobacterium alkalithermotolerans
WP 011019189.1 FtsZ Methanopyrus kandleri
TEU14147.1 FtsZ Hadesarchaea archaeon
KUO39120.1 FtsZ Hadesarchaea archaeon DG-33
KUO40134.1 FtsZ Candidatus Hadarchaeum yellowstonense
KUO42256.1 FtsZ Hadesarchaea archaeon YNP N21
WP 056933183.1 FtsZ Thermococcus barophilus
WP 011250372.1 FtsZ1 Thermococcus kodakarensis
ADE02725.1 FtsZ1 Haloferax volcanii DS2
WP 086975524.1 FtsZ1 Archaeoglobus fulgidus
RIB35621.1 FtsZ Candidatus Nanoclepta minutus
WP 010900500.1 FtsZ Thermoplasma acidophilum
WP 010869869.1 FtsZ1 Methanocaldococcus jannaschii
QQG39270.1 FtsZ Candidatus Woesearchaeota archaeon
RSN73625.1 FtsZ Candidatus Methanodesulfokora washburnensis
RDD53436.1 FtsZ Candidatus Korarchaeota archaeon NZ13-K
WP 125742028.1 FtsZ Candidatus Korarchaeum cryptofilum
ACB08089.1 FtsZ Candidatus Korarchaeum cryptofilum OPF8
