# Supplementary material for: Distinct filament morphology and membrane tethering features of the dual FtsZ paralogs in Odinarchaeota
Source: EMBO J. 2025 Aug 8;44(21):4. doi: 10.1038/s44318-025-00529-7 (PMC12583498; doi:10.1038/s44318-025-00529-7)
Supplement: Supplementary file 5 [file 44318_2025_529_MOESM7_ESM.zip › Expanded view Figures/Figure EV5/Fig. EV5/Fig. EV5.pdf]

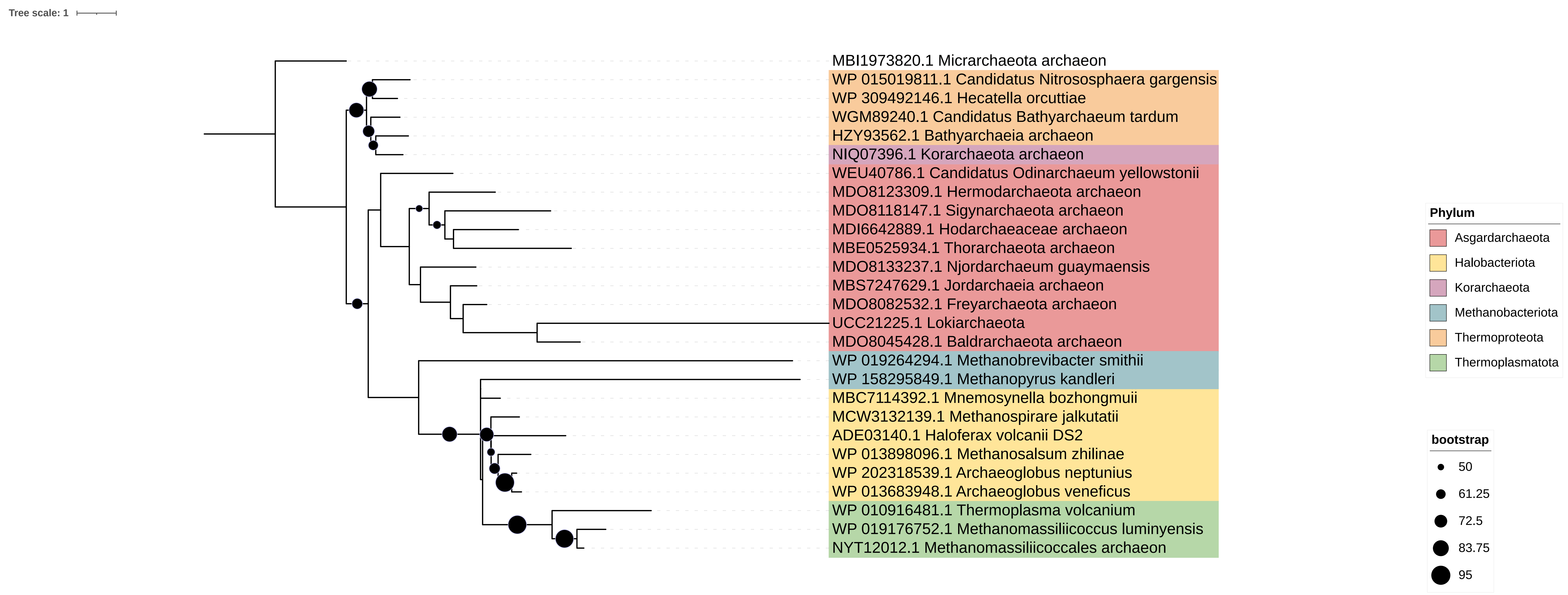
Tree scale: 1
MBI1973820.1 Micrarchaeota archaeon
WP 015019811.1 Candidatus Nitrososphaera gargensis
WP 309492146.1 Hecatella orcuttiae
WGM89240.1 Candidatus Bathyarchaeum tardum
HZY93562.1 Bathyarchaeia archaeon
NIQ07396.1 Korarchaeota archaeon
WEU40786.1 Candidatus Odinarchaeum yellowstonii
MDO8123309.1 Hermodarchaeota archaeon
MDO8118147.1 Sigynarchaeota archaeon
MDI6642889.1 Hodarchaeaceae archaeon
MBE0525934.1 Thorarchaeota archaeon
MDO8133237.1 Njordarchaeum guaymaensis
MBS7247629.1 Jordarchaeia archaeon
MDO8082532.1 Freyarchaeota archaeon
UCC21225.1 Lokiarchaeota
MDO8045428.1 Baldrarchaeota archaeon
WP 019264294.1 Methanobrevibacter smithii
WP 158295849.1 Methanopyrus kandleri
MBC7114392.1 Mnemosynella bozhongmuii
MCW3132139.1 Methanospirare jalkutatii
ADE03140.1 Haloferax volcanii DS2
WP 013898096.1 Methanosalsum zhilinae
WP 202318539.1 Archaeoglobus neptunius
WP 013683948.1 Archaeoglobus veneficus
WP 010916481.1 Thermoplasma volcanium
WP 019176752.1 Methanomassiliicoccus luminyensis
NYT12012.1 Methanomassiliicoccales archaeon
Phylum
Asgardarchaeota
Halobacteriota
Korarchaeota
Methanobacteriota
Thermoproteota
Thermoplasmatota
bootstrap
50
61.25
72.5
83.75
95
